# Whole Genome Sequences, De Novo Assembly, and Annotation of Antibiotic Resistant *Campylobacter jejuni* Strains S27, S33, and S36 Newly Isolated from Chicken Meat

**DOI:** 10.3390/microorganisms12010159

**Published:** 2024-01-13

**Authors:** Yiping He, Siddhartha Kanrar, Sue Reed, Joe Lee, Joseph Capobianco

**Affiliations:** Characterization and Interventions for Foodborne Pathogens Research Unit, Eastern Regional Research Center, Agricultural Research Service (ARS), United States Department of Agriculture (USDA), 600 East Mermaid Lane, Wyndmoor, PA 19038, USA; yiping.he@usda.gov (Y.H.); siddhartha.kanrar@usda.gov (S.K.); bugsue@ptd.net (S.R.); joe.lee@usda.gov (J.L.)

**Keywords:** *Campylobacter*, whole genome sequencing (WGS), assembly, annotation, foodborne pathogen

## Abstract

*Campylobacter* is a leading bacterial cause of gastrointestinal infections in humans and has imposed substantial medical and public health burdens worldwide. Among a total of 39 species in the *Campylobacter* genus, *C. jejuni* is the most important species responsible for approx. 90% of human *Campylobacter* illness. Most cases of the infection were acquired by ingesting undercooked poultry meat due to the high prevalence of *Campylobacter* in the products. Here, we reported the dataset of raw sequences, de novo assembled and annotated genomes of *C. jejuni* strains S27, S33, and S36 recently isolated from retail chicken by using PacBio highly accurate long-read sequencing technology combined with bioinformatics tools. Our data revealed several virulence and antibiotic resistance genes in each of the chromosomes, a type IV secretion system in the plasmid (pCjS33) of *C. jejuni* S33, and a type VI secretion system and a phage in the plasmid (pCjS36) of *C. jejuni* S36. This study not only provides new sequence data but also extends the knowledge pertaining to the genomic and functional aspects of this important foodborne pathogen, including the genetic determinants of virulence and antibiotic resistance.

## 1. Introduction

*Campylobacter* spp. are Gram-negative, spiral-shaped, highly motile bacteria which can cause human diseases such as campylobacteriosis, a form of gastroenteritis characterized by diarrhea, fever, abdominal pain, and nausea [1,2,3]. *Campylobacter* is the most common bacterial cause of foodborne illness in the world, responsible for an estimated 96 million cases annually [4], where 80–90% of human illnesses are due to *Campylobacter jejuni* [5]. The main source of infection is the consumption of contaminated raw or undercooked meat products, particularly poultry [5,6,7] due to the high prevalence of *Campylobacter jejuni* in retail chicken [8,9]. Studies have shown that *Campylobacter* can colonize the gastrointestinal tract of birds, livestock, and other animals without causing symptoms [10]. However, in humans, *Campylobacter* can invade the intestinal mucosa and trigger an inflammatory response, leading to tissue damage and fluid loss [11]. In some cases, *Campylobacter* infection can also result in serious complications, such as reactive arthritis, Guillain–Barré syndrome, and bacteremia [12].

Given the global burden and public health impact of *Campylobacter* infection, there is a need for a better understanding of the biology, diversity, and pathogenicity of this bacterium. Whole genome sequencing (WGS) is a powerful tool capable of providing comprehensive information regarding the genetic features and evolutionary relationships of foodborne pathogens such as *Campylobacter* [13]. WGS unveils the presence and distribution of genes associated with virulence, antibiotic resistance, secretion systems, and mobile genetic elements such as phages and plasmids. These elements may influence the survival, adaptation, and virulence of *Campylobacter* in diverse environments and hosts [14]. Furthermore, WGS facilitates the identification and characterization of novel *Campylobacter* strains, along with detecting and identifying outbreaks and transmission sources [15]. Therefore, WGS plays a crucial role in developing more effective and targeted strategies for preventing, diagnosing and treating *Campylobacter* infection [16].

In this study, the complete genome sequences and annotation of three *Campylobacter jejuni* strains (S27, S33, and S36) isolated from retail chicken in the United States are presented. An analysis was conducted on their genomic features in comparison to other available *C. jejuni* strains in public databases addressing their potential implications for food safety and public health. This is the first report of the complete genome sequences and annotation of *C. jejuni* strains S27, S33, and S36. These findings stand as valuable resources poised to facilitate future studies in comparative and functional genomics of this important foodborne pathogen.

## 2. Materials and Methods

### 2.1. Sample Preparation

*C. jejuni* strains S27, S33, and S36 were isolated from separate packages of raw chicken samples collected from local supermarkets using a previously described method [17]. Briefly, a 450 g chicken sample was rinsed with 250 mL Buffered Peptone Water, BPW (BioRad, Hercules, Ca). The rinse was concentrated by centrifugation and enriched microaerobically (5% O_2_, 10% CO_2_ and 85% N_2_) in Bolton broth (Oxoid, Basingstoke, Hampshire, UK) containing 5% laked horse blood (Remel, Lenexa, KS) and antibiotic selective supplement package containing cefoperazone, trimethoprim, vancomycin, and cycloheximide (Oxoid, Basingstoke, Hampshire, UK) at 42 °C for 24 h. Due to the high motility of *Campylobacter*, passive filtration with 0.65μm sterile cellulose acetate membranes (Merck-Millipore LTD, Cork Ireland) of the enrichment onto Brucella agar (Becton Dickinson, Sparks, MD) was chosen for bacterial isolation. Following colony purification, the genus and species of the isolates were determined using the previously reported multiplex qPCR assay [18]. Genomic DNA extraction was performed using the Qiagen genomic tip 100/G kit (Valencia, CA, USA) and quantified using the Qubit 3.0 fluorimeter (Thermo Fisher Scientific, Waltham, MA, USA) following the manufacturers’ instructions.

### 2.2. Whole Genome Sequencing, De Novo Assembly, and Annotation

Whole genome sequencing was conducted in the Sequel II Single Molecule Real Time (SMRT) system (Pacific Biosciences, Menlo Park, CA, USA). The library was constructed using the SMRTbell Prep Kit with the selection of insert sizes ranging from 500 bp to over 20 kb.

The raw reads obtained from PacBio long and accurate HiFi sequencing were deposited in the SRA database in GenBank. Subsequently, they underwent de novo assembly using Canu v2.2 [19] and were trimmed using the “getfasta” command of bedtools software v2.27.1. The sequences were then oriented to the *dnaA* starting point using Circlator 1.5.5 [20]. Functional annotation was carried out using the Rapid Annotation using Subsystem Technology (RAST) server [21]. All software was used with default parameters unless otherwise noted. The complete genomes were submitted to the genome database in GenBank and annotated using the NCBI Prokaryotic Genomic Annotation Pipeline (https://www.ncbi.nlm.nih.gov/genome/annotation_prok/, accessed on 7 August 2023) and the Rapid Annotation using Subsystems Technology (RAST, http://rast.nmpdr.org/, accessed on 27 July 2023).

## 3. Results

*C. jejuni* S27, S33, and S36 were isolated from separate packages of fresh chicken collected from local retailers in February 2023. These isolates displayed colonies with round shapes, smooth edges, and a glistening translucent yellowish or pinkish color on Brucellar agar plates. Confirmation of the genus and species of the strains was achieved through a real-time qPCR assay. Whole genome sequencing of *C. jejuni* S27, S33, and S36 from chicken samples was performed using PacBio long and accurate HiFi sequencing, followed by de novo assembly into complete genomes. Table 1 summarizes the statistics of raw sequence data and assembled complete genomes of the *C. jejuni* isolates.

The assembled genome sizes (~1.6–1.7 Mb) and G + C contents (30.4–30.5%) of these new isolates as shown in Table 1, align closely with other *C. jejuni* genomes found in the NCBI database. Each strain contains a circular chromosome and, in addition to this, *C. jejuni* S33 harbors a 40.7 kb plasmid (pCjS33), while *C. jejuni* S36 carries an 86.8 kb plasmid (pCjS36). Comparison with the available plasmids in GenBank showed that pCjS33 shares 99.30% sequence identity and 96% query coverage with the *Campylobacter* pTet plasmid, while pCjS36 exhibits up to 99.87% sequence identity and 98% query coverage with multiple *C. jejuni* plasmids.

The functional prediction of the chromosomes by Rapid Annotation using Subsystems Technology (RAST, http://rast.nmpdr.org/, accessed on 27 July 2023) is summarized in Table 2. Among the subsystems identified by RAST, there were more than 47 genes associated with the virulence, disease causation, defense, and motility of the *C. jejuni* strains, including *cadF*, *jlpA*, *porA*, and *pebA* genes associated with adhesion, *ciaB*, *pldA*, and *flaC* for invasion, and a cytolethal distending toxin *cdtABC* gene cluster.

The annotation of the pTet plasmid (pCjS33) in *C. jejuni* S33 revealed a gene cluster containing the *cag* pathogenicity island, which encodes a type IV secretion system (T4SS) and a *tetO* gene that encodes tetracycline resistance protein. This protein protects bacterial ribosomes from binding tetracycline. The prevalence of pTet family plasmids in *Campylobacter* is notable because they can be horizontally transmitted between the strains, which is a major factor in acquired resistance in *Campylobacter* spp. In contrast, the plasmid pCjS36 possesses a gene cluster associated with a type VI secretion system (T6SS), which is a novel virulence factor responsible for delivering toxic effectors. These effectors play roles in host colonization, cell adhesion and invasion, and the lysis of erythrocytes [22,23,24]. Collectively these findings underscore the potential of the strains to cause human disease.

Furthermore analyses of the *C. jejuni* S33 and S36 genomes revealed the presence of numerous phage proteins as predicted by the NCBI Prokaryotic Genomic Annotation Pipeline (PGAP) [25]. Table A1 and Table A2 show the genes and proteins associated with phage packaging, portals, and terminases in *C. jejuni* S33 and S36, respectively. Consistent with the PGAP predictions, PHASTER, a web-based phage search tool (https://phaster.ca/), predicted an intact 40 kb phage (30.58% GC content) containing 62 proteins along with phage attachment sites (*attL* and *attR)* within the sequence region 79591–119622 in *C. jejuni* S33 chromosome. Similarly, PHASTER detected a 43.4 kb intact phage (29.69% GC content) containing 56 proteins and phage attachment sites (*attL* and *attR*) within the sequence region 49017–92474 in *C. jejuni* S36. Bacteriophage and plasmids play pivotal roles in the horizontal transfer of genetic material. The finding of these mobile genetic elements within the genomes indicates genetic divergence and rearrangement in *Campylobacter* evolution.

## 4. Discussion

*Campylobacter jejuni* is a major cause of foodborne gastroenteritis worldwide, mainly associated with the consumption of contaminated poultry products. Despite this significance, the complete molecular mechanism behind *Campylobacter* infections remains incompletely understood, suggesting dependance on a number of virulence factors involving cell adhesion, invasion, and motility [26]. Whole genome sequencing was employed to examine the genomic characteristics of three *C. jejuni* strains (S27, S33, and S36) recently isolated from retail chicken in the United States. Comparing their genomic features with those of other *C. jejuni* strains available in public databases revealed the presence of virulence determinants within these three strains. These include factors associated with motility (*flaAB*, *flaC*, *flgE*, *flgP*, *flgR*, *flgS*, *fliS*, *fliW*, and *pflAB*) and chemotaxis (*cheA*, *cheW*, *cheV*, *cheY*, *cheR*, and cheB), as well as factors associated with adhesion and invasion to host cells (*htrA*, *cadF*, *flpA*, *jlpA*, *capA*, *porA*, *pebA*, *ciaAB*, and *pldA*). Furthermore, these strains carry cytolethal distending toxin (*cdtABC*) associated with binding to host cells, resulting in enlargement and cell death. Additionally, the strains possess lipooligosaccharide (LOS) facilitating attachment and endocytosis into host cells.

Additionally, a type IV secretion system was identified in the plasmid (pCjS33) of *C. jejuni* S33, a critical virulence factor typically encoded in mobile genomic islands (plasmids, conjugative elements, or pathogenicity islands). This system is involved in protein transfer across the cell envelope, enhances the oxidative stress response, and contributes to host colonization [27]. In contrast, the plasmid (pCjS36) of *C. jejuni* S36 contains a gene cluster that encodes a type VI secretion system. This system has demonstrated important roles in contact-dependent host cell adherence and invasion, promoting colonization, inducing cytotoxicity of red blood cells, and enhancing survival within the host gastrointestinal tract under conditions of oxidative stress [28,29].

The presence of these genes within the genomes could confer advantages to *C. jejuni*, enhancing its survival, adaptation, transmission, and pathogenicity across different environments and hosts. These findings emphasize the potential risks associated with *C. jejuni* infection originating from retail chicken, underscoring the need for improved food safety and public health measures. Moreover, this study contributes to understanding the molecular mechanisms governing *C. jejuni*–host interactions and horizontal gene transfer. Such insights may facilitate the development of novel therapeutic strategies for managing campylobacteriosis. Notably, this is the first report detailing the complete genome sequences and annotation of *C. jejuni* strains S27, S33, and S36. These resources hold value for future studies in comparative and functional genomics concerning this important foodborne pathogen.

## Figures and Tables

**Table 1:** Statistics of the sequence data and assembled genomes for *C. jejuni* strains.

*C. jejuni* Strain	SRA Accession No.	Accession No. Chromosome/ Plasmid	No. of Reads/ Av. Length	Quality	Reads N50/ N90	Average Read Depth	Size of Chromosome/Plasmid (bp)	GC Content of Chromosome/Plasmid (%)
S27	SRX21182642	CP131444/N/A	432,285/ 12,361	Q36	12,878/ 9041	3176	1,663,226/ N/A	30.5/ N/A
S33	SRX21182643	CP131442/CP131443	438,906/ 13,482	Q35	14,143/ 9886	699	1,748,761/ 40,686	30.4/ 28.4
S36	SRX21182644	CP131440/CP131441	420,379/ 12,137	Q36	12,672/8767	2768	1,715,845/ 86,827	30.4/ 26.0

**Table 2:** Summarized features of the annotated chromosomes in *C. jejuni* strains.

*C. jejuni* Strain	No. of Coding Sequences	No. of RNAs	No. of Functional Subsystems	No. of Virulence, Disease & Defense Genes	No. of Motility & Chemotaxis Genes
S27	1667	53	189	17	30
S33	1850	53	188	17	32
S36	1825	50	188	17	33

## Data Availability

Genome sequence reads were obtained from the PacBio Sequel II system. The raw sequences of *C. jejuni* strains S27, S33, and S36 were deposited into the SRA database in GenBank, NCBI under the identifiers of SRA: SRP451999 and Bioproject: PRJNA999693. The complete genome sequences (chromosome/plasmid) are available in GenBank under accession numbers CP131444, CP131442/CP131443, and CP131440/CP131441.

## Appendix A

**Table A1:** Phage proteins in *C. jejuni* S33 chromosome predicted by PGAP.

Locus_Tag in NCBI	Gene		Protein ID	Length of Amino Acid	Function
	Start	Stop			
Q7259_00400	85998	85510	WLF63783	162	Phage virion morphogenesis protein
Q7259_00405	88215	86002	WLF63784	737	Phage tail tape measure protein
Q7259_00415	88913	88674	WLF63786	79	Phage tail assembly protein
Q7259_00420	89575	89066	WLF63787	169	Phage major tail tube protein
Q7259_00425	90795	89602	WLF63788	397	Phage tail sheath family protein
Q7259_00445	93884	92805	WLF63792	359	Phage tail protein
Q7259_00450	94504	93884	WLF63793	206	Phage tail protein I
Q7259_00455	95667	94501	WLF63794	388	Phage baseplate J/gp47 family protein
Q7259_00460	95954	95664	WLF63795	96	Phage baseplate wedge protein/gp25 family protein
Q7259_00470	96783	96151	WLF63797	210	Phage baseplate assembly protein V
Q7259_00475	97097	96783	WLF63798	104	Phage holin family protein
Q7259_00505	98779	99594	WLF63804	271	Phage protease
Q7259_00515	100127	101104	WLF63806	325	Phage major capsid protein
Q7259_00530	102269	103939	WLF63809	556	Phage terminase large subunit
Q7259_00535	103949	105319	WLF63810	456	DUF935 family protein (Mu phage gp29)
Q7259_00540	105321	106559	WLF63811	412	Phage minor head protein
Q7259_00545	106685	107059	WLF63812	124	Phage tail protein
Q7259_00550	107052	107243	WLF63813	63	Phage tail protein X
Q7259_00555	107237	108214	WLF63814	325	Phage tail protein
Q7259_00570	109560	109276	WLF63817	94	Mor transcription activator family protein, phage Mu
Q7259_00585	111088	110639	WLF63819	149	Regulatory protein GemA, phage Mu
Q7259_00620	114570	114085	WLF63826	161	Host-nuclease inhibitor Gam family protein, phage
Q7259_00640	116413	115490	WLF63830	307	ATPase/bacteriophage DNA transposition B protein
Q7259_00645	118659	116584	WLF63831	691	Transposase family protein, phage Mu

**Table A2:** Phage proteins in *C. jejuni* S36 chromosome predicted by PGAP.

Locus_Tag in NCBI	Gene		Protein ID	Length of Amino Acid	Function
	Start	Stop			
Q7260_00275	59549	58572	WLF67118	325	Phage tail formation protein GpD
Q7260_00285	60101	59727	WLF67120	124	Phage tail protein GpU
Q7260_00290	61465	60227	WLF67121	412	Phage minor head protein (Mu phage gp30)
Q7260_00295	62837	61467	WLF67122	456	DUF935 family protein (Mu phage gp29)
Q7260_00300	64517	62847	WLF67916	556	Phage terminase large subunit
Q7260_00305	65116	64517	WLF67123	199	DUF1804 family protein (Mu phage gp31)
Q7260_00310	65567	65109	WLF67124	152	DUF1320 family protein (Mu phage gp36)
Q7260_00315	66660	65683	WLF67125	325	Major capsid protein E, phage head
Q7260_00325	68008	67193	WLF67127	271	Phage protease (Mu phage gp32)
Q7260_00355	69691	70005	WLF67133	104	Phage holin family protein
Q7260_00360	70005	70637	WLF67917	210	Phage baseplate assembly protein GpV
Q7260_00370	70834	71124	WLF67135	96	Phage baseplate assembly protein GpW (gp25 family)
Q7260_00375	71121	72287	WLF67136	388	Phage baseplate assembly protein GpJ (gp47 family)
Q7260_00380	72284	72904	WLF67137	206	Phage tail formation protein GpI
Q7260_00405	75918	77111	WLF67141	397	Phage tail sheath family protein
Q7260_00410	77138	77647	WLF67142	169	Phage major tail tube protein
Q7260_00415	77800	78039	WLF67143	79	Phage tail assembly protein
Q7260_00425	78498	80720	WLF67145	740	Phage tail tape measure protein
Q7260_00430	80724	81212	WLF67146	162	Phage virion morphogenesis protein
Q7260_00435	81317	82132	WLF67147	271	Phage DNA adenine methylase
Q7260_00455	83761	83132	WLF67151	209	S24 family peptidase (putative phage repressor protein)
Q7260_06585	1250342	1251517	WLF66625	391	Tyrosine-type recombinase/integrase (Phage integrase)
Q7260_06660	1258222	1257491	WLF66640	243	phage regulatory protein/anti-repressor Ant
Q7260_06750	1270310	1269993	WLF66658	105	head-tail adaptor protein
Q7260_06755	1270760	1270323	WLF66659	145	Phage gp6-like head-tail connector protein
Q7260_06765	1272185	1271019	WLF66661	388	Phage major capsid protein, HK97 family
Q7260_06770	1272759	1272202	WLF66662	185	HK97 family phage prohead protease
Q7260_06810	1282537	1281995	WLF66670	180	HK97 gp10 family phage protein
Q7260_06815	1283706	1282534	WLF66671	390	Phage portal protein
Q7260_06825	1285980	1284355	WLF66673	541	Phage terminase large subunit
Q7260_06830	1286619	1285984	WLF66674	211	P27 family phage terminase small subunit
